# Ovarioleukodystrophy Due to EIF2B Genes: Systematic Review and Case Report

**DOI:** 10.7759/cureus.64497

**Published:** 2024-07-13

**Authors:** Mariana Escobar-Pacheco, Mariana Luna-Álvarez, David Dávila-Ortiz de Montellano, Petra Yescas-Gómez, Miguel Á Ramírez-García

**Affiliations:** 1 Genetics, National Institute of Neurology and Neurosurgery Manuel Velasco Suárez, Mexico City, MEX; 2 Genetics, National Institute of Pediatrics, Mexico City, MEX

**Keywords:** ovarioleukodystrophy, mri imaging, vanishing white matter disease, eif2b1-5 genes, systematic review

## Abstract

Leukodystrophies comprise a spectrum of genetic disorders affecting white matter (WM) formation in the central nervous system (CNS), of which vanishing white matter disease (VWMD) is one. VWMD presents with progressive neurological deterioration and a variety of manifestations. Ovarioleukodystrophy, a subtype of VWMD, exhibits a distinctive clinical profile encompassing both CNS WM alterations and ovarian dysfunction. Variants in genes of the eukaryotic translation initiation factor 2B (EIF2B) complex affect the full form and are implicated in VWMD, including ovarioleukodystrophy. This work aimed to systematically review all published cases of ovarioleukodystrophy associated with variants in the *EIF2B1-5* gene complex based on the first case identified in a Mexican population.

We performed a systematic review according to the Preferred Reporting Items for Systematic reviews and Meta-Analyses (PRISMA) guidelines of published cases of ovarioleukodystrophy associated with the *EIF2B* gene complex, including a newly identified case from Mexico. We identified 207 publications using PUBMED, SCOPUS, and PMC databases. One hundred fifty-one publications were eliminated due to duplicates, titles, abstracts, or other reasons, while 56 publications were revised, of which 29 were eliminated because they dealt with other genes or non-human research, and 27 reports were assessed for eligibility. Finally, 14 reports describing ovarian involvement, neuroimaging, and molecular variants were included.

Our review identified 20 cases worldwide, with a median age of onset of 19 years. Clinical features included WM involvement, ovarian abnormalities, gait disturbances, epilepsy, cognitive and language impairment, and other neurological manifestations. Neuroimaging showed characteristic WM changes, highlighting the importance of MRI in diagnosis. Missense variants predominated among the identified genetic mutations, especially in the *EIF2B4* and *EIF2B5* genes.

Ovarioleukodystrophy is an ultra-rare disorder with a wide range of clinical manifestations and ovarian changes. Gynecological evaluation is crucial in suspected cases of ovarioleukodystrophy, as ovarian manifestations may precede neurological symptoms. The role of MRI is crucial in the diagnostic approach to this entity. Continued collaborative efforts are essential to elucidate genotype-phenotype correlations, improve clinical management, and promote therapeutic advances for this rare disorder.

## Introduction and background

Leukodystrophies are a group of genetic disorders characterized by altered formation of the white matter (WM) of the central nervous system (CNS) [1]. The age of onset varies widely, the clinical course is progressive, and there is currently no curative treatment [2]. Within this group of pathologies, we find vanishing white matter disease (VWMD), also known as childhood ataxia with central nervous system hypomyelination (CACH) [3]. This disease is clinically characterized by progressive neurological deterioration associated with a wide range of cognitive, psychiatric, and motor manifestations (spasticity, ataxia, etc.). In some cases, optic atrophy with visual loss has been reported. In addition, worsening of symptoms is observed after cranioencephalic injury or infection, with partial recovery [3, 4].

Manifestations are not limited to the CNS, as growth retardation, cataracts, hepatosplenomegaly, pancreatitis, renal hypoplasia, and ovarian dysfunction have also been reported (Table 1) [3]. The ovarian dysfunction most commonly presents as premature ovarian failure (cessation of menses before the age of 40 years and elevated serum follicle-stimulating hormone (FSH) levels of >40 U/L) [5]; this association has been termed ovarioleukodystrophy [5, 6].

**Table 1:** Clinical characteristics of the prenatal and adult stages of the VWMD VWMD - vanishing white matter disease

Prenatal	Aggressive clinical course: Oligohydramnios, decreased fetal movement, microcephaly, arthrogryposis, intrauterine growth restriction, hepatosplenomegaly, renal hypoplasia, pancreatitis, and cataracts; and in females, ovarian dysgenesis.
Postnatal (childhood and adult)	Variable clinical manifestations: Epilepsy, migraine, developmental delay, ataxia, spasticity, cognitive impairment, and psychiatric symptoms. Female patients may present with primary amenorrhea or premature ovarian failure.

VWMD is caused by genetic variants in any of the genes of the EIF2B complex (*EIF2B1*, *EIF2B2*, *EIF2B3*, *EIF2B4*, and *EIF2B5*), which encode the α, β, ɣ, δ and ɛ subunits respectively. These subunits form the eukaryotic translation initiation factor *EIF2B*(1-5), which has essential functions in initiating and controlling protein synthesis [7-9].

This work aimed to perform a systematic review of all published cases of ovarioleukodystrophy associated with variants in the *EIF2B* gene complex regarding the first case identified in a Mexican population.

## Review

Methodology

Case Description: Clinical Findings

A 34-year-old woman from Mexico City, the first child of a non-consanguineous marriage, was the product of a normal pregnancy. At the age of 13, she began to have frequent falls, not associated with weakness or other apparent changes. Around age 25, her relatives noticed that she had a gait disturbance (dragging of the left foot). There was also a feeling of stiffness in the left leg when bending the hip. She had also noticed a decrease in the frequency of her menstrual cycles since the age of 22, and she has had annual opsomenorrhea annually since the age of 24. Since 2020, she has been evaluated at our center for gait disturbances; however, she considers herself functional and independent in all activities of daily living. Recently, she had sporadic urinary incontinence. 

The MRI showed extensive hyperintensities in the bilateral and symmetrical subcortical WM, involving both cerebellar hemispheres and the brainstem. Her pelvic USG showed an undersized uterus (47x22x30 mm), and the ovaries could not be visualized (possibly due to their small size). Hormone levels were prolactin 8.68 ng/mL, FSH 38.20 mIU/mL (⭡), luteinizing hormone (LH) 23.30 mIU/mL, estradiol <5 ng/mL (⭣), and progesterone 0.2287 ng/mL. At her last visit (November 2023), she had a Montreal Cognitive Assessment (MoCA) test of 24 points; eye movements with hypometric saccades; spasticity in the right and left lower limbs, Ashworth Scale score 2 and 3, respectively; strength ⅘ in the left pelvic limb and preserved for the rest of the body; generalized hyperreflexia; Tromner and Hoffman signs in the left hemibody; bilateral plantar flexor response; upper limb dysmetria; and spastic gait and decreased right brachiation. Currently, she is being treated with levonorgestrel/ethinyl estradiol, calcium, and zoledronate.

Case Description: Genetic Analysis

A commercial molecular panel for leukodystrophies by NGS sequencing identified the variant *c.725C>T* (*NM_015636.3*; *p.Pro242Leu*) in the *EIF2B4 *gene in a homozygous state, a variant previously classified as likely pathogenic, confirming the diagnosis of VWMD with fibrodysplasia ossificans progressiva (FOP) or ovarioleukodystrophy. The variant segregation study identified both parents as carriers.

Systematic Review of All Published Cases

**Figure 1:**
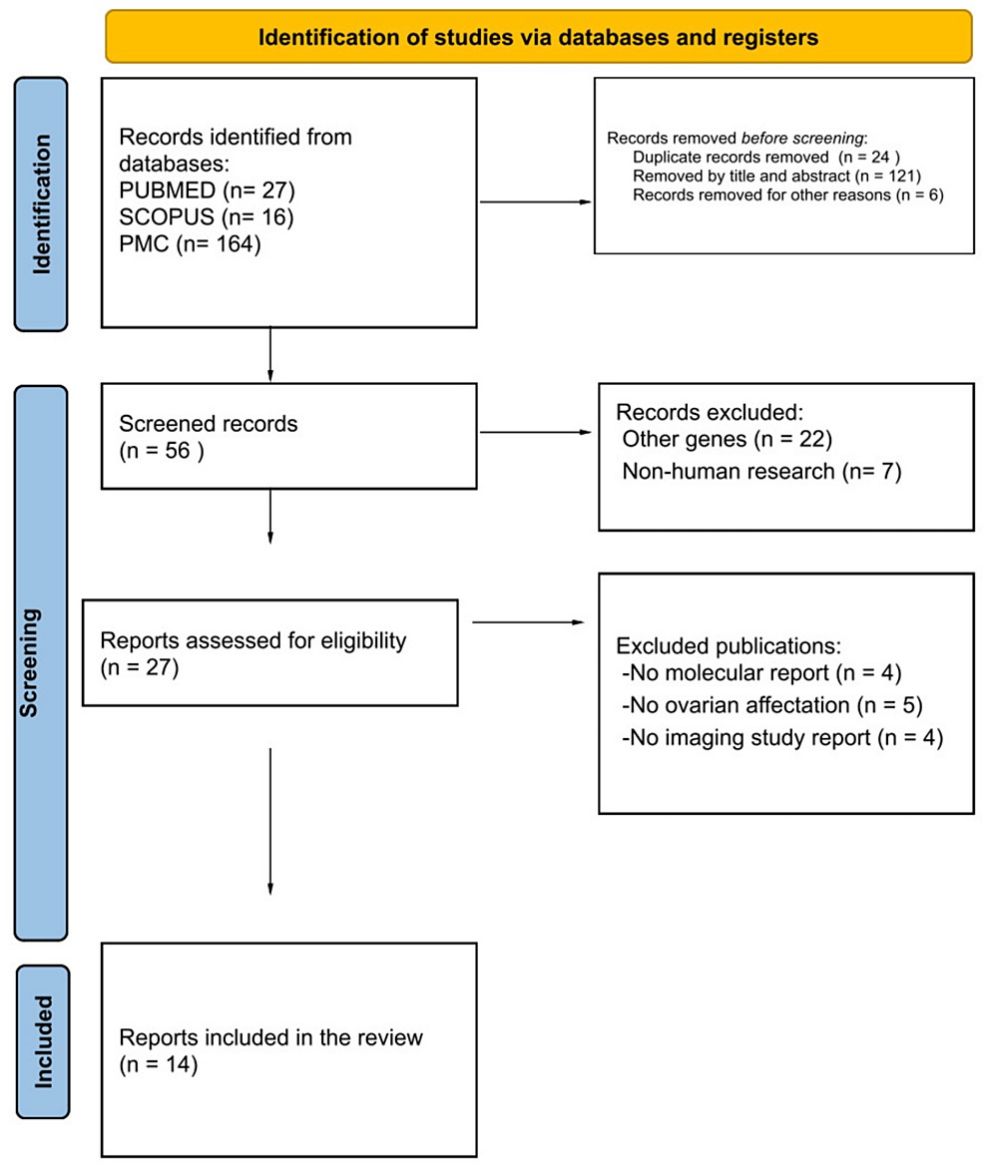
Flowchart of the search strategy

A literature search was conducted using systematic review procedures according to the PRISMA guidelines (Figure 1) [10]. Scopus, MEDLINE (via PubMed), and PubMed Central databases were searched from January 2003 (first published case) to July 2023. The combination of Medical Subject Headings (MeSH) terms 'ovarioleukodystrophy', 'leukodystrophy', 'ovarian failure', and '*EIF2B*(1-5)' (for each of the genes independently) was used. English was the only language filter used. 

The search prioritized the inclusion of any publication (controlled studies, cohorts, case series, case reports, reviews, or editorials) describing female subjects with the phenotype of interest, with a description of the molecular variant and a neuroimaging study (selection criteria). We excluded 1) articles that did not focus on the population of interest, 2) publications that did not focus on humans (animal or in vitro studies), or 3) publications for which the full text was not available. The references of the selected articles were consulted as additional information (snowball sampling method). 

We extracted the following information: author, year of publication, title, presentation, age of onset, cognitive changes, pyramidal, extrapyramidal, cerebellar, or psychiatric symptoms, other motor changes, neuroimaging findings, and molecular assessment. 

Results

Literature Review and Clinical Phenotype

After the literature search, 14 publications were selected, corresponding to nine case reports and five case series. Information from the identified patient was also included. This resulted in a series of 20 cases of women with ovarioleukodystrophy due to variants in the *EIF2B *gene complex. The median age of onset was 19 years (range 0.6-40). The clinical features present in the entire sample were WM involvement and ovarian changes, the latter corresponding to ovarian dysgenesis (5%), primary amenorrhea (15%), secondary amenorrhea (15%), and premature ovarian failure (60%). In addition, 45% had gait disturbances and pyramidal manifestations. 35% had epilepsy, and one-third had cognitive and language impairment, extrapyramidal manifestations, and cerebellar signs. The remaining manifestations are described in Table 2.

**Table 2:** Clinical characteristics in subjects with ovarioleukodystrophy

Characteristics	% (n=20)
Age of onset	
Prenatal	5% (1/20)
Neonatal	0% (0/20)
Childhood	15% (3/20)
Adolescence	20% (4/20)
Adulthood	60% (12/20)
Developmental delay	25% (5/20)
Cognitive impairment	45% (9/20)
Memory impairment	25% (5/20)
Attention impairment	35% (7/20)
Language impairment	20% (4/20)
Visuospatial impairment	45% (9/20)
Pyramidal signs	45% (9/20)
Gait disturbance	35% (7/20)
Epilepsy	30% (6/20)
Extrapyramidal signs	25% (5/20)
Altered eye movements	20% (4/20)
Sphincter dysfunction	30% (6/20)
Psychiatric manifestations	35% (7/20)
Cerebellar signs	10% (2/20)
Muscle tone	15% (3/20)
Strength	15% (3/20)
Ophthalmological alterations	15% (3/20)
Sensory alterations	20% (4/20)
Feeding disorders	25% (5/20)
Headache	25% (5/20)
Abnormal imaging findings	100% (20/20)
Ovarian disorders	100% (20/20)

Neuroimaging Findings

**Figure 2:**
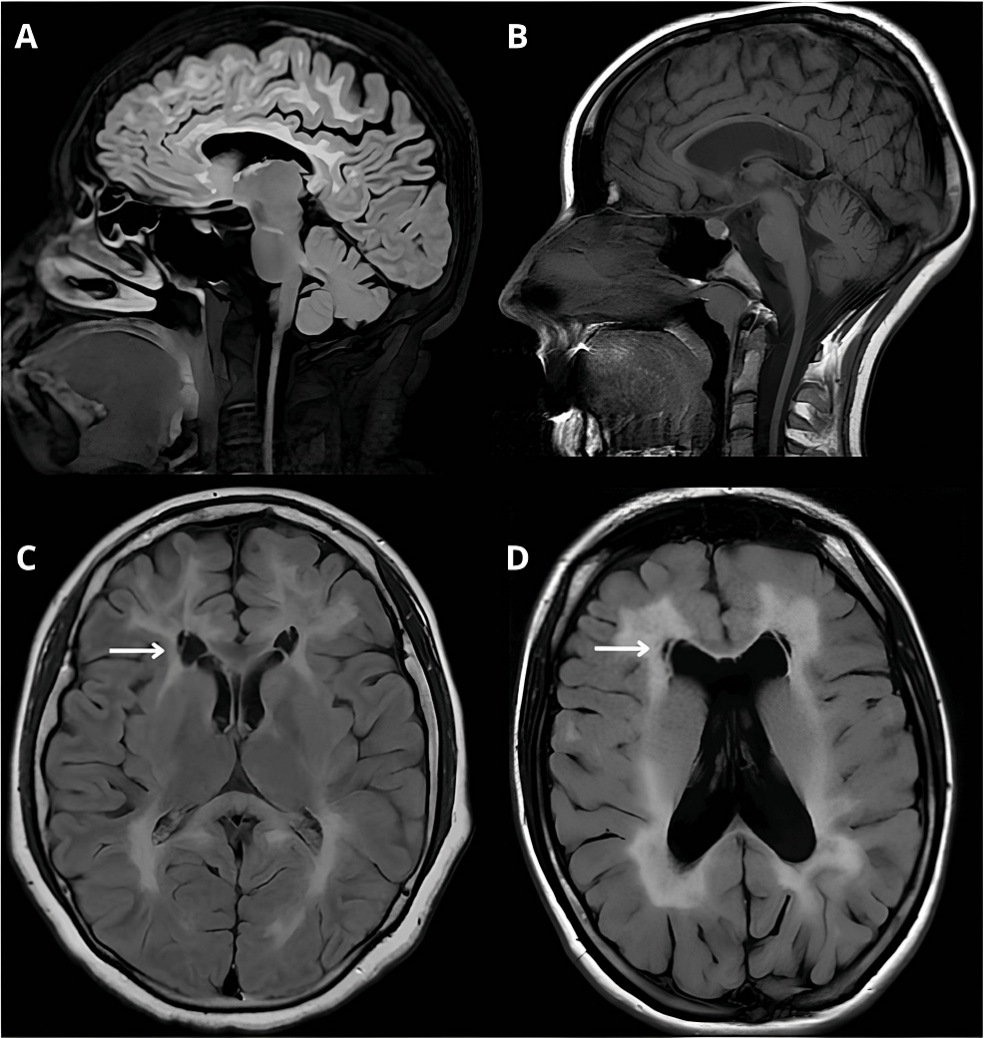
Neuroimaging findings in ovarioleukodystrophy due to alterations in the EIF2B gene complex A) Parasagittal T2 FLAIR-weighted image of the patient identified in this report showing subcortical hyperintensities in the WM, generalized cortico-subcortical atrophy, and significant atrophy of the corpus callosum, and C) axial T2 FLAIR-weighted image of the same patient showing periventricular hyperintensities and cystic degeneration of the WM at the level of the frontal horns (arrow). B) and D) similar findings with generalized and corpus callosum atrophy and cystic degeneration, as reported by Rodríguez-Palmero et al. (reproduced under a CC-BY license) [22].

Neuroimaging changes associated with VWMD are diverse and mainly show WM involvement, characterized by T2 hyperintense and T1 hypointense lesions with diffuse periventricular distribution. The brainstem and cerebellum may be involved. WM involvement is independent of age (e.g., case one vs. case 14, Table 3). There is also generalized cortical and corpus callosum atrophy. In addition, a very characteristic finding is a cystic degeneration of the WM at the level of the frontal horns (Figure 2). Table 3 describes the imaging findings in the 20 cases identified.

**Table 3:** Imaging findings. ch - compound heterozygote; h - homozygote; MRI - magnetic resonance imaging; MRI T2-FLAIR - fluid-attenuated inversion recovery; WM - white matter

Author	Year	Case and genotype	Neuroimaging findings
van der Knaap et al. [11]	2003	Case 1, *EIF2B4* c.1172C>A(h)	MRI T2-FLAIR with white matter lower signal intensity, consistent with rarefaction; widening of the gyri; white matter with normal signal intensity for unmyelinated white matter on T2; ventricles laterals are slightly dilated. T2 MRI at 5 months shows important atrophy of the cerebral white matter with wide dilation of the lateral ventricles and marked atrophy of the cerebellum.
Ohlenbusch et al. [12]	2005	Case 2, *EIF2B1*: c.547G>T (h)	MRI with hyperintensities on T2 and hypointensities on T1 in the spinal field.
		Case 3, *EIF2B1*: c.547G>T (h)	MRI with hyperintensities on T2 and hypointensities on T1 in the spinal field.
		Case 4, *EIF2B2*:c.512C>T and c.599G>T(ch)	MRI with distinct symmetrically medullary demyelination.
		Case 5, *EIF2B4*: c.806T>G and c.1120C>T (ch)	MRI with abnormal symmetrical and diffuse signal of the cerebral hemispheric white matter and atrophy of the cortex and cerebellum.
		Case 6, *EIF2B5*: c.338G>A and c.929G>T (ch)	MRI with abnormal symmetrical and diffuse signal of the white matter and generalized atrophy.
Mathisa et al. [13]	2008	Case 7, *EIF2B5*: c.338G>A (h)	MRI with symmetric hyperintensity of the cerebral hemispheric white matter on FLAIR and T2 bilateral areas of cavitation in the parieto-occipital region, and atrophy of the cerebral hemispheres and corpus callosum.
Imam et al. [14]	2011	Case 8, *EIF2B5*: c.338G>A and c.896G>A (ch)	MRI with hypointensities in frontal periventricular white matter.
La Piana et al. [15]	2012	Case 9, *EIF2B3*: c.260C>T andc.272G>A (ch)	MRI with diffuse leukoencephalopathy in the brain and cerebellum, and bilateral cystic areas in the frontal horns in FLAIR.
Robinson et al. [16]	2014	Case 10, *EIF2B3*: c.260C>T and c.272G>A (ch).	MRI with diffuse hyperintensities on T2, hypointensities on T1, and cystic areas in the frontal horns.
Ibitoye et al. [17]	2016	Case 11, *EIF2B5*: c.869>A and c.913A>T (ch)	MRI with changes consistent with leukopathy and restricted diffusion in the right occipital lobe.
Mukerji et al. [18]	2016	Case 12, *EIF2B2*: c.818A>G (h)	MRI T2-FLAIR with hyperintensities in white matter and periventricular cavitations.
Herrera-García et al. [19]	2017	Case 13, *EIF2B4*: c.1117C>T (h)	MRI T2 with hyperintensities in supratentorial white matter and microcysts adjacent to the frontal horns.
		Case 14, *EIF2B4*: c.1117C>T (h)	MRI at 13y without specific white matter lesions. MRI at 37y with generalized and symmetrical white matter hyperintensities, with rarefaction and predominant cystic areas in semioval centers on T2.
Zhang et al. [20]	2017	Case 15, *EIF2B5*: c.1759A>G	T2-weighted images show diffuse, symmetric WM abnormalities, with suspicion of cavitation. FLAIR images show diffuse WM lesions with mixed-signal and cavitation; the low intensity suggests intense WM damage and rarefaction. T1-weighted and diffuse-weighted show lesions in the splenium and genu of the corpus callosum.
Tucker et al. [21]	2018	Case 16, *EIF2B2*: c.514C>T and [c.818A>G (ch)	MRI with focal regions of gliosis and encephalomalacia in the right medial frontal (previous cerebral infarction) and confluent areas of white matter abnormalities, adjacent to the lateral ventricles.
Rodriguez-Palmero et al. [22]	2020	Case 17, *EIF2B5*: c.725A>G and c.1156+13G>A (ch)	MRI T2 with hyperintensities in periventricular and pons white matter; Cortico-subcortical, spinal, and corpus callosum atrophy.
Parihar et al. [23]	2022	Case 18, *EIF2B3*: c.687T>G (h)	MRI with diffuse and symmetrical leukoencephalopathy.
Flores et al. [24]	2023	Case 19, *EIF2B5*: c.338G>A	MRI shows subcortical and periventricular WM lesions that appear hypointense on T1-weighted images and hyperintense on T2-weighted and FLAIR. FLAIR images showed cystic degeneration within diffuse hyperintense WM involvement.
This publication	2024	Case 20, *EIF2B4*: c.725C>T (h)	MRI T2-FLAIR with diffuse increased signal in the subcortical, deep, and periventricular white matter; cystic encephalomalacia of the white matter adjacent to the frontal recesses and in the corona radiata and superior frontal gyri bilaterally; generalized cortico-subcortical and corpus callosum atrophy.

Pathogenic Variants in Genes of the EIF2B Complex Associated With Ovarioleukodistrophy 

**Figure 3:**
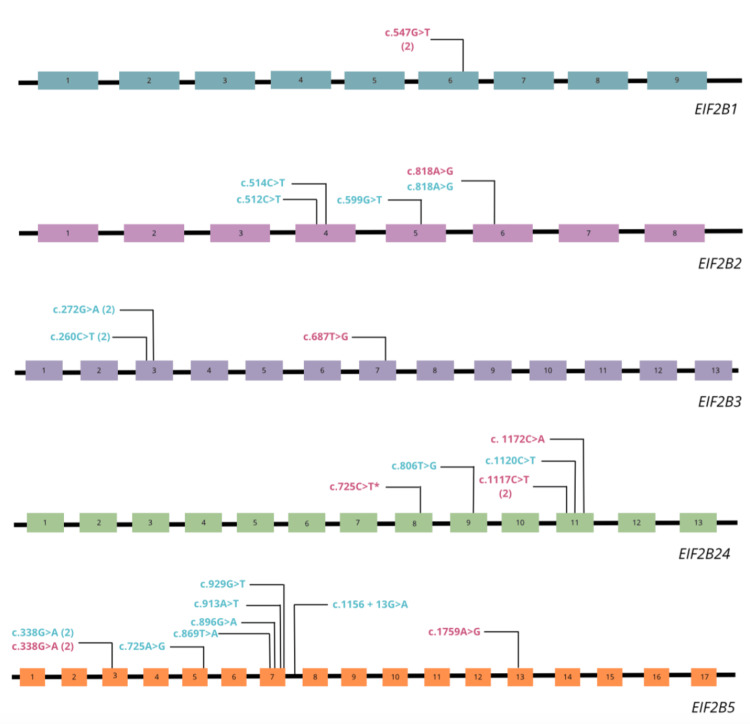
Schematic representation of the identified variants in the EIF2B1-5 gene complex Homozygous variants are shown in pink, and compound heterozygotes are shown in blue. The patient variant reported in this publication is marked with *.

The highest proportion of cases was due to variants in the *EIF2B4* (five cases) and *EIF2B5* (seven cases) genes, followed by the *EIF2B2* and *EIF2B3* genes with cases each and only two cases for the *EIF2B1* gene (the clinical description for each gene is detailed in Table 4). All cases were autosomal recessive, 50% were homozygous, and most variants were missense (see Table 3). Figure 3 shows the distribution of the variants for each gene.

**Table 4:** Main clinical characteristics by affected gene

Characteristics	EIF2B1	EIF2B2	EIF2B3	EIF2B4	EIF2B5
Affected subjects	(n=2)	(n=3)	(n=3)	(n=5)	(n=7)
Age of onset					
Prenatal	0/2	0/3	0/3	1/5	0/7
Neonatal	0/2	0/3	0/3	0/5	0/7
Childhood	0/2	0/3	0/3	1/5	2/7
Adolescence	2/2	0/3	0/3	3/5	0/7
Adulthood	0/2	3/3	3/3	0/5	5/7
Developmental delay	2/2	0/3	0/3	2/5	1/7
Cognitive impairment	2/2	0/3	2/3	1/5	2/7
Memory impairment	2/2	1/3	1/3	1/5	1/7
Attention impairment	1/2	1/3	0/3	1/5	1/7
Language impairment	2/2	0/3	1/3	2/5	2/7
Visuospatial impairment	0/2	1/3	1/3	1/5	1/7
Pyramidal signs	0/2	0/3	1/3	3/5	6/7
Gait disturbance	0/2	1/3	1/3	3/5	5/7
Epilepsy	0/2	1/3	0/3	4/5	2/7
Extrapyramidal signs	0/2	1/3	0/3	2/5	5/7
Altered eye movements	0/2	0/3	0/3	2/5	4/7
Sphincter dysfunction	0/2	0/3	1/3	2/5	1/7
Psychiatric manifestations	2/2	0/3	2/3	1/5	1/7
Cerebellar signs	0/2	1/3	1/3	2/5	4/7
Muscle tone	0/2	0/3	0/3	1/5	1/7
Strength	0/2	0/3	1/3	1/5	2/7
Ophthalmological alterations	0/2	0/3	0/3	1/5	2/7
Sensory alterations	0/2	0/3	2/3	0/5	1/7
Feeding disorders	0/2	0/3	0/3	3/5	2/7
Headache	2/2	0/3	2/3	0/5	1/7
Abnormal imaging findings	2/2	3/3	3/3	5/5	7/7
Ovarian disorder	2/2	3/3	3/3	5/5	7/7
Dysgenesis	-	-	-	1	-
Structural alteration	-	-	-	-	-
Primary amenorrhea	1	2	-	1	-
Secondary amenorrhea	1	-	-	-	2
Premature ovarian failure	-	1	3	3	5
Primary infertility	-	-	-	-	-

Discussion

Ovarioleukodystrophies are a broad group of pathologies with central nervous system (CNS)-WM involvement and ovarian disorders showing wide phenotypic variability. Several causative genes have been identified (*EIF2B* gene complex, *AARS2*, *LARS2*, *KARS*, and the genes associated with Perrault syndrome). Variants in the *EIF2B* gene family causing VWMD correspond to the first pathologies related to the development of ovarioleukodystrophy [11-24]. It seems that the simultaneous involvement of the cerebral WM and ovaries could be explained by the significant expression of these genes (*EIF2B1*, *EIF2B3*, *EIF2B4*, *EIF2B5*, and *AARS2*) in these tissues according to the GTExPortal database. 

According to our review, ovarioleukodystrophy should be broadened beyond FOP alone, as the clinical manifestations and ovarian changes are diverse. Gait disturbances and pyramidal manifestations are common, as was the case in our patient, who was initially diagnosed with spastic paraparesis. Cognitive and language deficits, extrapyramidal manifestations, and cerebellar signs were seen in one-third of the cases. The age of onset is also highly variable, with the earliest identified case being a fetal presentation and more than half having an adult onset. However, the proportion of cases with adolescent-onset is not negligible, so ovarioleukodystrophy should be considered in adolescent and prepubertal females with WM disorders, as the absence of noticeable gynecologic changes may mask the diagnosis. Therefore, we believe that all women presenting with leukoencephalopathy/ leukodystrophy, regardless of age, should be evaluated gynecologically to rule out ovarian manifestations.

The role of MRI is crucial in the diagnostic approach to this entity and all leukoencephalopathies/leukodystrophies. In these pathologies, the suspicion is mainly based on the pattern of WM involvement in neuroimaging. Consistent with this, in this group of cases of ovarioleukodystrophy, WM involvement shows a pattern of global periventricular involvement that increases with progression and may show cerebellar involvement. In addition, a prominent imaging finding in this entity is cystic degeneration at the level of the frontal horns, which was present in 45% of the sample and is due to progressive rarefaction and cystic degeneration of the affected WM, which is subsequently replaced by fluid [3]. 

Regarding variants in the *EIF2B1-5* genes, most variants associated with ovarioleukodystrophy were missense variants, similar to those associated with classic VWMD [6]. The *EIF2B *complex plays a crucial role in the regulation of protein synthesis through a mechanism of phosphorylation of the transcription factor eIF2; the phosphorylated eIF2 binds to the initiator tRNA in association with ribosomes and promotes recognition of the mRNA start codon AUG, ensuring the correct initiation of protein synthesis of each mRNA [7]. However, the small proportion of identified cases of ovarian leukodystrophy due to variants in the *EIF2B1-5* genes makes it difficult to establish a genotype-phenotype relationship. However, qualitatively, we observed that the cases associated with the *EIF2B4* gene showed the most extended age of onset compared to the other genes, as in the case of our patient, with onset in adolescence and a progressive course with a predominance of the pyramidal syndrome. On the other hand, because the subjects of ovarioleukodystrophy are so rare, it is challenging to define mutation hotspots in the studied genes associated with this phenotype. Most variants affect exonic regions, except an intronic variant identified in the *EIF2B5* gene. As more cases of this pathology and their related variants are described, we should determine whether studying intronic regions in these genes is necessary for the diagnostic approach. 

Ovarioleukodystrophy is an ultra-rare entity, and, to our knowledge, only 20 cases associated with variants in the *EIF2B1-5* genes have been described worldwide through this systematic review. However, the molecular approach is crucial because it allows a concrete diagnostic definition. Although there are no specific treatments for this entity, proper identification allows clinical follow-up to improve quality of life, develop more accurate sequencing panels, and generate targeted therapies [25]. The current treatment approach for VWD consists of symptomatic and preventive management of situations that may exacerbate the condition, such as infections and head trauma, as recovery is often partial or absent [3]. Some treatments under investigation for VWD focus on viral vectors, induced pluripotent stems (iPSCs), and even genomic editing, such as CRISPR-Cas9 [25-27]. As the understanding of ovarioleukodystrophy improves, more personalized treatments are expected.

## Conclusions

VWMD is a very rare disorder, and the phenotype known as ovarioleukodystrophy could be considered an ultra-rare entity. Due to its recessive inheritance, it is essential to rule out consanguinity and/or inbreeding. This review included clinical data from 20 cases of ovarioleukodystrophy due to variants in the *EIF2B1-5* genes described worldwide, including one new case identified in Mexico. The most common ovarian alteration was premature ovarian failure, but hormonal and gynecologic evaluation is essential in all patients with suspected leukoencephalopathy/leukodystrophy. Although most cases have occurred in adulthood, early-onset forms (including prenatal onset) should be noticed. It is essential to continue to identify and share molecular and clinical information on patients with this extremely rare diagnosis to provide genotype-phenotype correlations for better clinical follow-up, genetic counseling and prognosis, and the opportunity to develop specific treatments.
